# Central Administration of Recombinant IGF1 Is Neuroprotective in a Rodent Model of Acute Liver Failure

**DOI:** 10.3390/ijms27083547

**Published:** 2026-04-16

**Authors:** Yubo Wang, Matthew McMillin, Gabriel Frampton, Kathryn Rhodes, Elaina Williams, Juliet Venter, Jace Tyson, Esha Gupta, Mihika Patankar, Patrick Mireles, Sharon DeMorrow

**Affiliations:** 1 Division of Pharmacology and Toxicology, College of Pharmacy, The University of Texas at Austin, Austin, TX 78712, USA; yubowang@utexas.edu (Y.W.); kathryn.rhodes@utexas.edu (K.R.); elainacoree@gmail.com (E.W.); juliet.venter@austin.utexas.edu (J.V.); jace.e.tyson@utexas.edu (J.T.); eshagupta@utexas.edu (E.G.); mihikapatankar@utexas.edu (M.P.); pr_mireles@utexas.edu (P.M.); 2 Department of Innovation, Education and Technology, Baylor College of Medicine, Temple, TX 76508, USA; matthew.mcmillin@bcm.edu; 3 Department of Neurosurgery, Dell Medical School, The University of Texas at Austin, Austin, TX 78712, USA; gabriel.frampton@austin.utexas.edu; 4 Department of Internal Medicine, Dell Medical School, The University of Texas at Austin, Austin, TX 78712, USA

**Keywords:** hepatic encephalopathy, neuroinflammation, insulin-like growth factor 1 (IGF1), microglia, azoxymethane, neuron–microglia communication

## Abstract

Acute liver failure is often accompanied by neurological disturbances collectively referred to as hepatic encephalopathy (HE), characterized by neuroinflammation and subsequent cognitive decline. Insulin-like growth factor 1 (IGF1) is a neuroprotective peptide with anti-inflammatory properties in the brain. The role of IGF1 in cognitive deficits and neuroinflammation during HE remains largely unexplored. In C57Bl/6 mice, HE was established through an intraperitoneal injection of azoxymethane (AOM), and tissues were collected at defined time points during disease development. IGF1 expression in the cortex was downregulated following AOM administration. Central infusion of recombinant mouse IGF1 (rmIGF1) before AOM injection resulted in delayed neurological impairment, reduced microglial activation, and decreased proinflammatory cytokine and chemokine production in AOM mice. In vitro, rmIGF1 and conditioned media derived from rmIGF1-treated primary neurons attenuated phagocytic activity and C–C motif chemokine ligand 2 (CCL2) production in the microglial cell line EOC-20. Collectively, our results show that IGF1, whose levels decline during HE, alleviates neuroinflammation and improves the pathological state of AOM-treated mice through the suppression of microglial activation and the regulation of neuron–microglia paracrine communication.

## 1. Introduction

Acute liver failure (ALF) is a severe condition characterized by rapid liver dysfunction, coagulopathy, and hepatic encephalopathy (HE) in individuals without pre-existing liver disease [1]. HE is a severe consequence of liver failure resulting in a range of cognitive deficits that heavily impact quality of life. Hyperammonemia is acknowledged as the main factor affecting brain metabolism and function, both directly and indirectly [2]. HE can occur in patients with normal ammonia levels, indicating that elevated ammonia is not the sole pathogenic factor [3]. Neuroinflammation, in conjunction with ammonia, is recognized as a crucial pathogenic element in HE, primarily mediated by glial cells like astrocytes, microglia, and oligodendrocytes [4,5]. Among these glial cells, microglia, the innate immune cells of the brain, act as crucial regulators of neuroinflammation and respond to damage, as well as acting as pathogenic drivers in neurodegenerative diseases [6]. Inflammatory markers that activate microglia include C–C motif chemokine ligands 2 and 5 (CCL2, CCL5), as well as C–X–C motif chemokine ligand 1 (CXCL1). Upon activation, microglia secrete proinflammatory cytokines and chemokines, which are known to trigger downstream local immune cell recruitment [7]. Neuronal release of CCL2, interleukin-1β (IL-1β), and tumor necrosis factor-α (TNF-α) is associated with subsequent reactive microgliosis [8]. Neuronal CCL2 is crucial for neuron–microglia communication, and disruptions in this signaling impair microglial and neuronal activity, neurotransmission, phagocytosis, synaptic pruning, and chemokine–receptor interactions, ultimately contributing to the development of HE [9]. Together, elevated systemic inflammation and circulating inflammatory molecules during liver damage may lead to the induction of neuroinflammation and encephalopathy [10].

Insulin-like growth factor 1 (IGF1), a 7.5 kDa polypeptide, acts as a neurotrophic factor in the central nervous system (CNS), contributing to neurogenesis, synapse formation, and neuronal excitability [11]. IGF1 functions as a neuroprotectant in vivo, offering protection against ischemic strokes associated with excessive proinflammatory cytokine production, as observed in conditions like stroke, brain trauma, and multiple sclerosis [12,13]. In all types of cells in the CNS, microglia contribute prominently to local IGF-1 production compared with astrocytes and neurons, while its receptor is predominantly found in neurons and astrocytes [14]. Moreover, IGF1 plays a role in neuroinflammatory responses. IGF1 mitigates inflammation by suppressing LPS-induced cytokine production in the brain, achieved through inhibiting glial activation and promoting brain-derived neurotrophic factor [12]. In the periphery, the liver predominantly produces IGF1, with its expression controlled by the growth hormone [11]. Despite numerous studies, it remains unclear how IGF1 regulates microglia–neuron communication and consequently shapes neuroinflammation during HE.

Accordingly, this study hypothesizes that the IGF1 level is decreased during HE, and restoration of IGF1 protein levels may attenuate HE-associated microglial activation by acting directly on microglia or indirectly through neuronal modulation, thus reducing neuroinflammation and improving neurological deficits during HE.

## 2. Results

### 2.1. IGF1 Is Downregulated in the Cortex of AOM-Treated Mice

**Figure 1:**
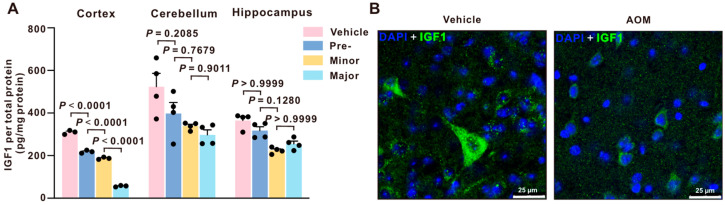
Selective and progressive loss of IGF1 expression in the cortex during ALF. (**A**) IGF1 levels as measured by ELISA in the cortex, cerebellum, and hippocampus from vehicle and time-course AOM-treated mice (n = 3–4). (**B**) Representative immunofluorescence images of cortical IGF1 immunofluorescence in mice treated with vehicle and AOM at the major time point. Scale bar = 25 µm. Data are presented as mean ± SEM, with individual animals shown as dots. The differences between multiple groups were analyzed using one-way ANOVA, followed by Tukey’s (normally distributed and equal variance) and the Kruskal–Wallis test, followed by Dunn’s multiple comparison test (not normally distributed). A *p*-value < 0.05 was statistically significant.

The regulation of IGF1 during HE was investigated by injecting mice with azoxymethane (AOM) and evaluating them at different stages of neurological impairment: before decline (Pre), with minor symptoms [15], and with major deficits (Major) [15]. IGF1 expression in the cortex significantly decreased following acute liver failure (ALF) before the development of overt neurological dysfunction and decreased significantly throughout the experiment. In comparison, IGF1 levels in the cerebellum showed a less robust pattern of change, while no significant or consistent alterations were detected in the hippocampus (Figure 1A). Therefore, subsequent studies were conducted with a focus on the cortex. Immunofluorescence images in the frontal cortex for IGF1 demonstrated that AOM-treated mice had a similar trend with a significant reduction in IGF1 immunoreactivity compared to vehicle (Figure 1B).

### 2.2. IGF1 Is Selectively Reduced in Microglia and Neurons

**Figure 2:**
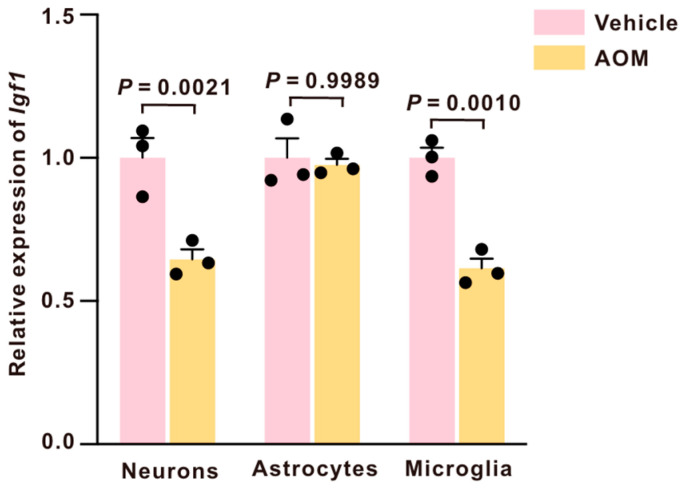
AOM selectively reduces *Igf1* mRNA expression in cortical neurons and microglia but not astrocytes. *Igf1* mRNA expression levels in cortex-derived neurons, astrocytes, and microglia. Data are presented as mean ± SEM, with individual animals shown as dots. The differences between multiple groups were analyzed using one-way ANOVA, followed by Tukey’s post hoc multiple comparison tests. A *p*-value < 0.05 was statistically significant.

Considering the pivotal functions of neurons, astrocytes, and microglia in cortical homeostasis and neuroinflammatory responses, we next assessed IGF1 expression in these cell populations following AOM treatment. In the cortex, qPCR analysis demonstrated a significant reduction in *Igf1* mRNA expression in neurons and microglia following AOM. In contrast, astrocytic *Igf1* expression remained unchanged (Figure 2), indicating cell-type-specific regulation of *Igf1* in the cortex.

### 2.3. Central Infusion of rmIGF1 Is Protective Against AOM-Induced Neurological Decline

**Figure 3:**
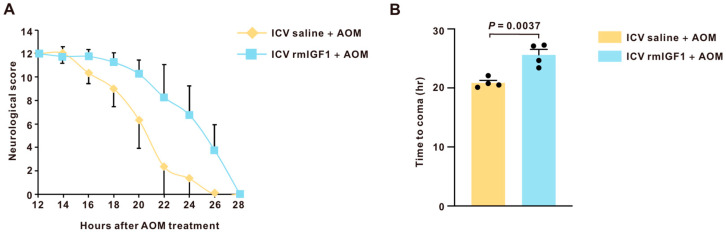
Central infusion of rmIGF1 provides neuroprotection in mice treated with AOM. (**A**) Graph depicting the neurological decline in mice treated with AOM compared to those treated with AOM + rmIGF1. The neurological score, comprising five reflex scores and an ataxia assessment as detailed in the methods, ranges from 0 (no reflex response) to 12 (normal reflex scores) (n = 4). (**B**) Time to coma in hours following AOM administration in mice treated with AOM and those treated with AOM + rmIGF1 (n = 4). Data are presented as mean ± SEM, with individual animals shown as dots. The difference between the two groups was analyzed using a two-tailed *t*-test. A *p*-value < 0.05 was statistically significant.

As IGF1 concentrations were decreased in the cortex after AOM treatment, the function of IGF1 in this model was further investigated. Central infusion of recombinant mouse IGF1 (rmIGF1) was found to markedly delay neurological decline compared with vehicle (Figure 3A). The duration to reach coma was significantly extended in rmIGF1-infused AOM-treated mice compared to saline-infused AOM-treated mice (Figure 3B).

### 2.4. Central Infusion of rmIGF1 Does Not Affect AOM-Induced Liver Injury

**Figure 4:**
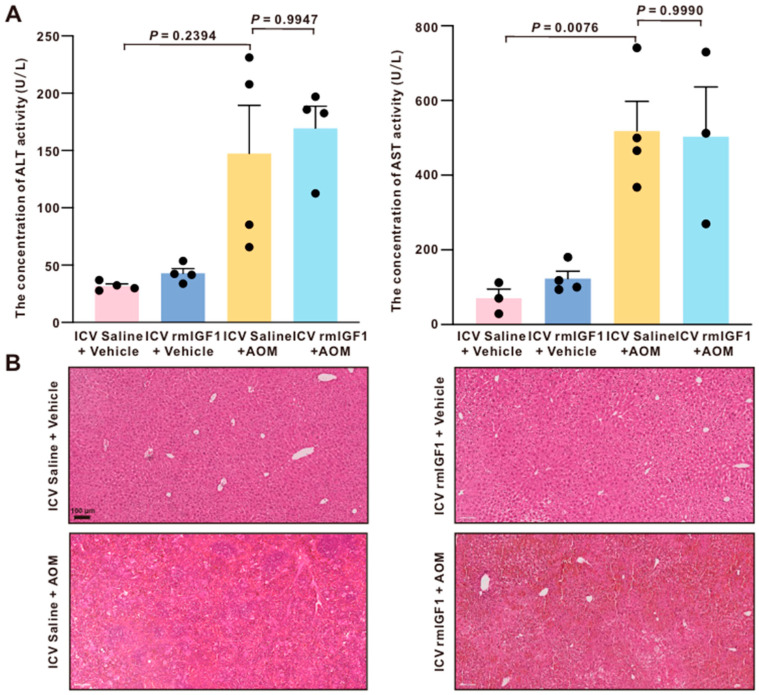
Central infusion of rmIGF1 does not alter AOM-induced liver injury. (**A**) Serum ALT and AST levels were measured in units per liter of serum in saline-infused or rmIGF1-infused mice treated with vehicle or AOM (n = 3–4). (**B**) H&E-stained liver sections from saline-infused or rmIGF1-infused mice treated with vehicle or AOM. Scale bar = 100 µm. Data are presented as mean ± SEM, with individual animals shown as dots. The differences between multiple groups were analyzed using one-way ANOVA, followed by Tukey’s (equal variance) post hoc multiple comparison tests, and Brown–Forsythe and Welch one-way ANOVA (not equal variance), followed by Games–Howell multiple comparison post hoc testing. A *p*-value < 0.05 was statistically significant.

To ensure that the neuroprotective role of rmIGF1 was not due to the remission of underlying liver injury, acute liver damage was assessed. Serum alanine transaminase (ALT) and aspartate aminotransferase (AST) activities are commonly used as biochemical indicators of liver injury [16]. AST and ALT levels were significantly elevated in saline-infused AOM-treated mice compared to control mice, with no notable difference between saline-infused and rmIGF1-infused AOM-treated groups (Figure 4A).

Liver histology corroborated the serum enzyme measurements. On H&E-stained sections, AOM administration induced prominent hepatic necrosis that was absent in both saline-treated controls, regardless of infusion condition. Moreover, no qualitative differences in necrosis—a hallmark of acute liver failure—or gross tissue morphology were observed between saline-infused and rmIGF1-infused mice following AOM treatment (Figure 4B).

### 2.5. Central Infusion of rmIGF1 Influences Microglia Morphology Without Changing Cell Density

**Figure 5:**
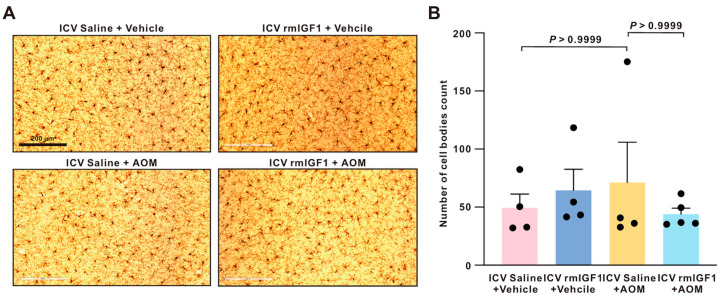
Central infusion of rmIGF1 does not alter microglial cell body density in the cortex following AOM treatment. (**A**) Representative cortical IBA1 immunohistochemical images. Scale bar = 200 µm. (**B**) Quantification of microglia cell bodies. Data are presented as mean ± SEM, with individual animals shown as dots. The differences between multiple groups were analyzed using the Kruskal–Wallis test, followed by Dunn’s multiple comparison test (not normally distributed). A *p*-value < 0.05 was statistically significant.

Considering *Igf1* mRNA expression decreased in cortical microglia and neurons after AOM treatment, we then determined whether IGF1 influences microglial cellular characteristics in the cortex following AOM treatment. Immunohistochemical assessment of IBA1 immunoreactivity was performed to investigate microglia in the cortex and to provide a broad-field analysis for the number of microglia present in a given region as a result of AOM injection. In the cortex, staining with IBA1 indicated that central infusion of rmIGF1 does not significantly alter the number of microglia in the cortex (Figure 5A,B).

**Figure 6:**
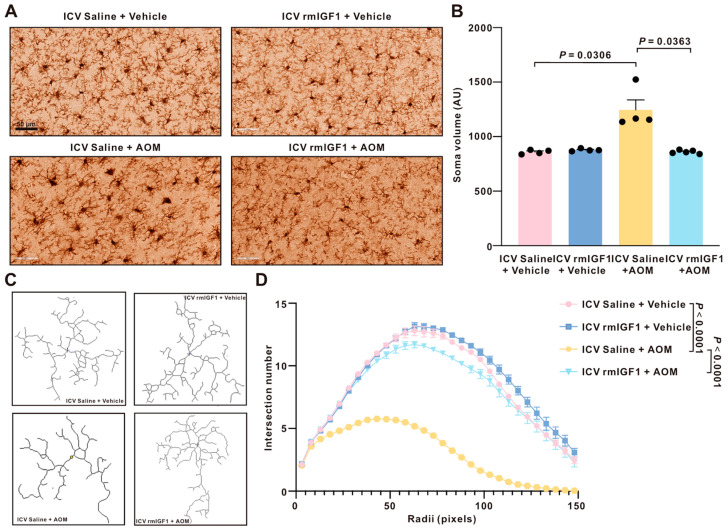
Central infusion of rmIGF1 attenuates AOM-induced microglia hypertrophy and preserves process complexity in the cortex. (**A**) Representative cortical IBA1 immunohistochemical images. Scale bar = 50 µm. (**B**) Quantification of microglial soma volume (AU). (**C**) Representative skeletonized tracings of individual microglia used for morphometric analyses. (**D**) Sholl analysis of microglial process complexity. Data are presented as mean ± SEM, with individual animals shown as dots. The differences in soma volume among groups were analyzed using the Kruskal–Wallis test, followed by Dunn’s multiple comparison test (not normally distributed). The differences in Sholl profiles among groups were analyzed using two-way ANOVA, with Factor A (treatment group) and Factor B (radius), followed by Tukey’s post hoc multiple comparison test. A *p*-value < 0.05 was statistically significant.

To determine if central infusion of rmIGF1 was involved in changes in microglia morphology, soma size and branch complexity were assessed on high-magnification images of Iba1 immunostaining (Figure 6A). The average soma size of the AOM mice was significantly increased compared to that of the control mice in the cortex. This was attenuated in the rmIGF1-infused AOM-treated mice (Figure 6B). Sholl analysis assessing branch complexity and retraction revealed a similar trend in changes to microglia morphology. Compared with saline-infused control mice, AOM significantly reduced process branching/complexity. However, this reduction can be attenuated by pretreatment with rmIGF1 (Figure 6C and Figure S1).

### 2.6. Central Infusion of rmIGF1 Attenuates Proinflammatory Cytokine and Chemokine Expression

To investigate whether the reduced microglial activation in rmIGF1-infused, AOM-treated mice reflected direct central actions of rmIGF1 or indirect modulation of cortical chemotactic cues, we quantified the mRNA expression of selected pro- and anti-inflammatory cytokines and chemokines in the cortex by qPCR.

**Figure 7:**
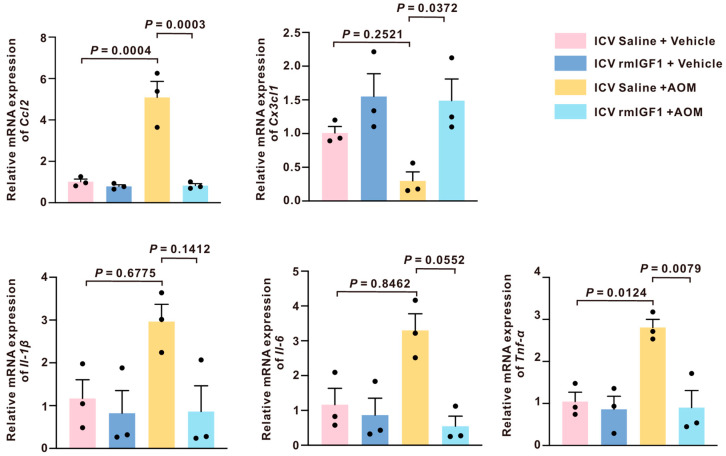
Central infusion of rmIGF1 selectively modulates AOM-induced inflammatory gene expression in the cortex. *Ccl2*, *Cx3cl2*, *Il-1β*, *Il-6*, and *Tnf-α* mRNA expression in the cortex of saline-infused or rmIGF1-infused mice treated with vehicle or AOM (n = 3). Data are presented as mean ± SEM, with individual animals shown as dots. The differences between multiple groups were analyzed using one-way ANOVA, followed by Tukey’s (normally distributed and equal variance), and the Kruskal–Wallis test, followed by Dunn’s multiple comparison test (not normally distributed). A *p*-value < 0.05 was statistically significant.

In AOM-induced HE, cortical CCL2 is upregulated and primarily localized to neurons [17]. This observation was replicated in our results, as cortical Ccl2 expression was markedly elevated in the saline-infused AOM-treated mice compared to the saline-infused controls. However, this was attenuated in the rmIGF1-infused AOM-treated mice (Figure 7). In contrast, fractalkine (CX3CL1) is predominantly neuronal and signals through microglial CX3CR1 to keep microglia in a less reactive state; this pathway is markedly suppressed in AOM-induced hepatic encephalopathy, and restoring fractalkine centrally mitigates neuroinflammation [18]. Here, cortical Cx3cl1 expression was downregulated in the saline-infused AOM-treated mice but was significantly restored by pretreatment with rmIGF1 (Figure 7).

As for proinflammatory cytokines, activated microglia amplify neuroinflammation by secreting key proinflammatory cytokines, including interleukin-1β (IL-1β), IL-6, and tumor necrosis factor alpha α (TNF-α) [8]. Here, cortical Il-1β, Il-6, and Tnf-α expression were all higher in the saline-infused AOM-treated mice compared to the saline-infused controls. However, only Tnf-α expression was significantly downregulated by pretreatment with rmIGF1 (Figure 7). In contrast, rmIGF1 did not significantly reduce Il-1β expression (ns; *p* = 0.1412) and only showed a modest, non-significant tendency to lower Il-6 (*p* = 0.0552).

### 2.7. rmIGF1 Directly and Neuron-Dependently Modulates Microglial Inflammatory Responses In Vitro

**Figure 8:**
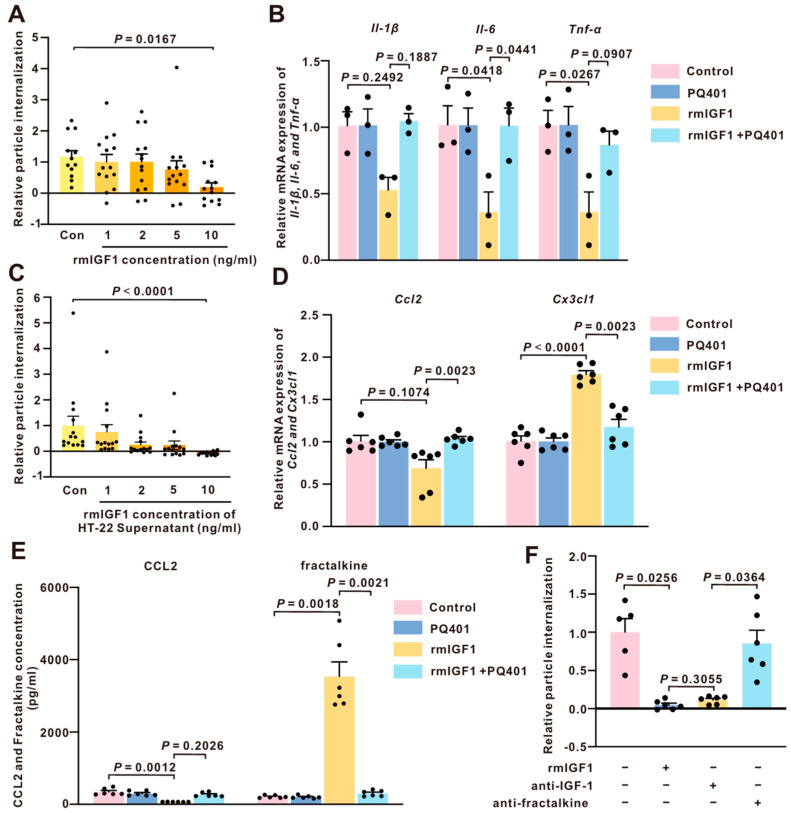
rmIGF1 inhibits microglial activation directly and via neuron-derived signals in vitro. (**A**) The study assessed relative phagocytosis in EOC-20 cells by measuring fluorescence from engulfed fluorescent E.coli bioparticles after treatment with varying concentrations of rmIGF1 (n = 12–14). (**B**) *Il-1β*, *Il-6*, and *Tnf-α* mRNA expression in EOC-20 cells treated with rmIGF1 at 10 ng/mL and IGF1R inhibitor PQ401 at 25 μM (n = 3). (**C**) The research also examined phagocytosis in EOC-20 cells exposed to conditioned media from HT-22 cells treated with different rmIGF1 concentrations (n = 14). (**D**) *Ccl2* and *Cx3cl2* mRNA expression in HT-22 cells treated with rmIGF1 at 10 ng/mL and IGF1R inhibitor PQ401 at 25 μM (n = 6). (**E**) ELISA analysis of CCL2 and fractalkine concentrations in conditioned media from HT-22 cells treated with rmIGF1 (10 ng/mL) in the presence or absence of the IGF1R inhibitor PQ401 (25 μM) (n = 6). (**F**) To determine whether fractalkine mediates the effects of rmIGF1-treated HT-22 conditioned media on microglial phagocytosis, neutralizing antibodies against IGF-1 or fractalkine were added to the conditioned media derived from rmIGF1-treated HT-22 cells. Phagocytic activity of EOC-20 cells was then assessed by measuring fluorescence from engulfed fluorescent *E. coli* bioparticles (n = 5–6). The differences between multiple groups were analyzed using one-way ANOVA, followed by Tukey’s (normally distributed and equal variance); Brown–Forsythe and Welch one-way ANOVA (normally distributed and not equal variance), followed by Games–Howell multiple comparison post hoc testing; and the Kruskal–Wallis test, followed by Dunn’s multiple comparison test (not normally distributed). A *p*-value < 0.05 was statistically significant.

To investigate the regulatory role of IGF1 in microglial activation in vitro, the EOC-20 cell, a mouse microglia cell line exhibiting phagocytic activity, was cultured and exposed to rmIGF1 and PQ401 (IGF1R inhibitor) for 4 h (Figure S2A). Treatment of EOC-20 cells with rmIGF1 at 10 ng/mL significantly reduced the phagocytosis activity of these cells (Figure 8A). qPCR data show that treatment with rmIGF1 at 10 ng/mL significantly reduced EOC-20 cell-secreted *Il-6* and *Tnf-α* levels, while *Il-1β* expression was not significantly altered. Notably, co-treatment with the IGF1R inhibitor PQ401 attenuated the rmIGF1-induced suppression of *Il6* and *Tnf-α*, suggesting an IGF1R-dependent effect.

To further investigate if the regulatory role of IGF1 in microglial activation is neuron-dependent, HT-22 cells, a mouse hippocampal neuron cell line, were cultured and exposed to rmIGF1 and PQ401 (Figure S2B). Treatment of EOC-20 cells with the conditioned media from HT-22 cells, which were treated with rmIGF1 at 10 ng/mL, significantly reduced the phagocytosis activity of EOC-20 cells (Figure 8C). Whether neuronal chemokine signaling, which regulates microglial responses, was altered by IGF1 was also investigated. The qPCR data showed that treatment with rmIGF1 at 10 ng/mL produced a modest reduction in *Ccl2* expression and a significant reduction in *Cx3cl2* expression in HT-22 cells. However, co-treatment with the IGF1R inhibitor PQ401 significantly attenuated both rmIGF1-induced downregulation of *Ccl2* and upregulation of *Cx3cl1* (Figure 8D). Consistent with the qPCR data, ELISA analysis of HT-22 conditioned media further demonstrated that rmIGF1 treatment markedly increased fractalkine secretion, whereas CCL2 levels changed only minimally. Importantly, co-treatment with the IGF1R inhibitor PQ401 significantly attenuated the rmIGF1-induced increase in fractalkine secretion (Figure 8E). To further determine whether the reduced phagocytic activity of EOC-20 cells was mediated by fractalkine secreted from IGF1-treated HT-22 cells, neutralizing antibodies against IGF-1 or fractalkine were added to the conditioned media derived from rmIGF1-treated HT-22 cells. Neutralization of IGF-1 did not restore the rmIGF1-induced reduction in EOC-20 phagocytic activity. In contrast, neutralization of fractalkine significantly rescued EOC-20 phagocytosis (Figure 8F), suggesting that the reduced phagocytic activity was mediated by HT-22-derived fractalkine rather than residual IGF-1 in the conditioned medium. These data indicate that IGF1 regulates microglial activation both directly and via neurons in vitro.

## 3. Discussion

Taken together, our data demonstrate that IGF1 signaling is a central modulator of cortical neuroinflammatory changes in the AOM-induced HE model. Cortical IGF1 expression decreased following AOM-induced ALF in a cell-type-dependent manner, with reductions observed in neurons and microglia. Importantly, central infusion of rmIGF1 altered cortical microglial activation and regulated neuroimmune communication by selectively shaping microglia-relevant inflammatory and chemokine pathways and by engaging both direct microglial and indirect neuron-mediated signaling. Collectively, these findings support a working model in which HE-associated loss of cortical IGF1 signaling contributes to maladaptive microglial activation, whereas restoring central IGF1 signaling constrains neuroinflammatory remodeling through coordinated microglia-intrinsic and neuron–microglia regulatory mechanisms.

In the CNS, IGF1 is produced in the hippocampus, cerebellum, hypothalamus, subventricular zone, and cerebral cortex [19]. In the cortex, IGF1 levels increase with an enriched environment, which primes visual cortex maturation [20]. In this study, we found a rapid loss of IGF1 expression in the cortex during AOM-induced HE, with IGF1 levels declining before overt neurological dysfunction and remaining suppressed as HE progressed. The cortex is often prominently involved in acute HE, potentially due to its high synaptic activity and energetic demand, which can render cortical circuits more sensitive to hyperammonemia and oxidative stress [21,22,23,24]. Suppression of IGF1 expression in the cortex is involved in the pathogenesis of AOM-induced HE [25]. The comparatively modest changes observed in the cerebellum and hippocampus may reflect regional differences in vulnerability to HE-related metabolic stress and neuroinflammatory signaling. Notably, cell-type-resolved analyses further showed that AOM reduced *Igf1* mRNA expression in cortical neurons and microglia, while astrocytic *Igf1* expression remained unchanged, indicating a cell-specific vulnerability of the neuron–microglia axis. This pattern is biologically plausible given that the liver is the principal source of circulating IGF1 and that hepatic dysfunction disrupts circulating IGF1 in the blood, leading to systemic IGF1 deficiency in the CNS [26,27]. Importantly, our findings extend and refine previous IGF1-related studies in the CNS and liver disease context. While IGF1 has been broadly recognized for its neuroprotective and anti-inflammatory roles, prior studies have largely focused on systemic IGF1 deficiency or peripheral administration, without resolving its cell-type-specific mechanisms within the brain during acute HE. In contrast, the present study demonstrates that IGF1 signaling is selectively disrupted within the cortex at early stages of AOM-induced HE and identifies a coordinated neuron–microglia regulatory axis as a key mechanistic target.

In this study, we found that central infusion of rmIGF1 is neuroprotective in AOM-induced HE by delaying neurological decline and prolonging the time to reach coma. A critical issue is whether the neuroprotective effects of central rmIGF1 reflect a true CNS mechanism or are secondary to reduced hepatotoxicity. In this study, serum ALT and AST levels were robustly increased by AOM yet did not differ between saline-infused and rmIGF1-infused AOM-treated mice, indicating that rmIGF1 infusion did not measurably ameliorate the primary hepatic injury. Consistent with these findings, liver histology showed comparable necrosis and gross morphology in AOM-treated animals regardless of infusion condition, further supporting the conclusion that rmIGF1 did not alter AOM-induced liver pathology. Together, these data suggest that the beneficial effects of rmIGF1 on neurological outcomes are not driven by hepatoprotection but rather reflect a central modulation of the brain’s response to acute liver failure, consistent with our prior work showing that CNS-targeted interventions can attenuate neurological decline even when liver injury remains largely unchanged [10]. The mechanisms responsible for the selective reduction in IGF-1 signaling during HE remain incompletely understood. Several factors associated with liver failure may contribute to this phenomenon. First, impaired hepatic function may disrupt the growth hormone–IGF-1 axis, leading to reduced systemic IGF-1 availability. Second, systemic inflammation and elevated circulating cytokines observed in HE may suppress IGF-1 production or signaling [28,29]. In addition, hyperammonemia, a key feature of HE, has been reported to interfere with endocrine and metabolic pathways that regulate neurotrophic signaling [30]. Further studies will be required to determine how these factors interact to regulate IGF-1 expression and signaling during HE progression.

Considering AOM-associated changes in *Igf1* expression occur predominantly in neurons and microglia, rather than astrocytes, highlights the neuron–microglia axis as a potential locus of IGF1-dependent regulation. IGF1 has been widely implicated in shaping neuroinflammatory tone, with reported roles in modulating cytokine signaling and glial reactivity, and microglia, the resident innate immune cell type of the brain, are recognized as central effectors of neuroinflammation across diverse CNS insults [12,31]. Therefore, we focused on microglial responses as a mechanistic readout of central IGF1 signaling in AOM-induced HE.

Microglial responses can manifest as changes in cell number/density (e.g., proliferation or recruitment) and as changes in activation state reflected by morphological remodeling. Microglial morphology is typically preceded by changes in microglial recruitment, indicating a reactive phenotype in the presence of damage [32]. In the AOM model, we observed no significant differences in microglial cell body counts/density across treatment groups, indicating that neither AOM nor central rmIGF1 infusion substantially altered microglial abundance in the cortex. In contrast, AOM induced pronounced morphological changes consistent with reactive microglia, including an increase in soma size, a retraction of branches, and thickening of processes among the branches. Importantly, central rmIGF1 infusion significantly attenuated these AOM-associated morphological abnormalities by reducing soma hypertrophy and partially restoring branching profiles toward the vehicle controls. Such a pattern is consistent with the concept that early neuroinflammatory responses in acute HE may be dominated by rapid phenotypic remodeling of resident microglia, whereas changes in microglial number can be variable, time-dependent, and model-specific [15,33].

Our results further indicate that central infusion of rmIGF1 altered the gene expression of both *Ccl2* and *Cx3cl1*, two chemokines that represent complementary facets of neuroimmune signaling. We previously found that in AOM-induced hepatic encephalopathy, cortical CCL2 is upregulated and primarily localized to neurons. Inhibition of CCR2/CCR4 signaling mitigates microglial activation and neurological decline, implicating CCL2 as a key upstream chemokine driving neuroinflammation [17]. CX3CL1 (fractalkine) is predominantly neuron-derived and functions as a key homeostatic regulator of microglial reactivity through CX3CR1 in response to brain injury or inflammation [34]. The simultaneous sensitivity of these two axes to rmIGF1 suggests that IGF1 signaling may recalibrate microglial tone by dampening a CCL2-linked “pro-reactive” cue while also adjusting the CX3CL1-dependent neuron-to-microglia restraint pathway, rather than simply suppressing inflammation globally. As for proinflammatory cytokines, rmIGF1 exerted a more pronounced effect on *Tnf-α* compared with *Il-6* and *Il-1β*. Elevations of the proinflammatory cytokines IL-1β, IL-6, and TNF-α are observed in both patients and rodent models of acute liver failure [35,36]. However, TNF-α is an upstream driver of other downstream neuroinflammatory signaling pathways, and, consistent with this role, TNF-α levels peak earlier than those of other cytokines following LPS stimulation during neuroinflammatory responses [37,38,39]. Moreover, TNF-α stimulation produces a distinct microglial morphology [40]. Interestingly, TNF-α, derived from activated microglia, functions as an autocrine mediator in microglial activation, inducing other inflammatory mediators and thereby promoting neuroinflammation [41]. Together, these data support a model in which IGF1 preferentially targets a chemokine-centered and TNF-dominant reactive module. Moreover, considering that reactive microgliosis may be driven by Purkinje neuron-derived TNF-α, which forms a feedback loop between microglia and neurons [42,43]. Therefore, the impact of IGF1 on the microglia and neuron loop needs to be further investigated.

To investigate whether IGF1 influences microglia directly or indirectly via neurons, we employed complementary in vitro paradigms using EOC-20 microglia and HT-22 neuron cell lines. Direct rmIGF1 exposure altered microglial functional and transcriptional readouts, including phagocytic activity and inflammatory gene expression, and these effects were blunted by pharmacological inhibition of IGF1R with PQ401, supporting a microglia-intrinsic pathway that is IGF1R-dependent. IGF-1 signals primarily via IGF-1 receptors (IGF-1R) but can also interact with the insulin receptor [14]. Notably, among the inflammatory transcripts examined, *Tnf-α* exhibited the most pronounced rmIGF1-dependent modulation, which is consistent with our in vivo findings showing a preferential effect of central rmIGF1 on TNF-α-associated inflammatory signaling. In parallel, conditioned media from rmIGF1-treated HT-22 cells significantly reduced particle internalization by EOC-20 microglia, indicating that rmIGF1 can also modulate microglial function indirectly through neurons. Consistent with this neuron-mediated mechanism, rmIGF1 altered neuronal chemokine expression—most notably increasing *Cx3cl1* in an IGF1R-dependent manner—providing a plausible signaling link between neuronal IGF1 responsiveness and downstream changes in microglial activation. Collectively, these experiments establish an in vitro “closed loop” in which IGF1 signaling engages both microglia-intrinsic pathways and neuron-to-microglia communication, offering a mechanistic framework that helps explain the selective chemokine and cytokine modulation and microglial phenotypic remodeling observed in vivo during AOM-induced HE. All in vitro studies used immortalized EOC-20 and HT-22 cell lines and pharmacological IGF1R inhibition (PQ401), which may not fully recapitulate primary cell behavior and can introduce off-target effects. Validation in primary microglia and neurons, as well as rodent models with genetic manipulation of IGF1 and IGF1R in a cell-type-specific manner, will be necessary to strengthen this conclusion. Our cell-type-resolved qPCR provides evidence for neuron- and microglia-associated changes on transcript-level readouts; future work should focus on incorporating protein quantification and spatial localization. Moreover, IGF-1 receptor signaling is known to activate several canonical downstream pathways, including the PI3K/Akt and MAPK signaling cascades, which regulate inflammatory signaling and cell survival in the central nervous system. These pathways have also been implicated in modulating microglial activation states [14]. Although the present study focused on the functional role of IGF-1 in neuron–microglia communication during hepatic encephalopathy, future studies will be required to determine the specific downstream signaling pathways involved in mediating these effects and determine how IGF-1 signaling interacts with other pathways involved in HE pathogenesis. By integrating in vivo and in vitro approaches, we demonstrate that IGF1 exerts both direct microglia-intrinsic effects and indirect neuron-mediated regulation of microglial function. This dual mechanism provides new insight into how neurotrophic signaling pathways orchestrate cell–cell communication during acute liver failure, which has not been clearly defined in previous IGF1 studies. Furthermore, unlike previous studies that primarily describe global anti-inflammatory effects of IGF1, our data reveal that IGF1 does not uniformly suppress neuroinflammation but instead selectively modulates specific chemokine pathways, including the CCL2 and CX3CL1 axes, alongside preferential regulation of TNF-α signaling. This highlights a more detailed role of IGF1 in reshaping neuroimmune communication rather than broadly inhibiting inflammatory responses.

Together, these findings position IGF1 not simply as a general neuroprotective factor but as a context-dependent regulator of neuron–microglia crosstalk during acute HE. However, the present findings are derived from an acute AOM-induced HE model, and determining the optimal therapeutic window, dose, and route for augmenting central IGF1 signaling, as well as evaluating efficacy across chronic HE models and in the context of other neuroprotective pathways, will be essential for assessing the translational perspective of IGF1.

## 4. Materials and Methods

### 4.1. Materials

Azoxymethane (AOM) was purchased from Sigma-Aldrich (Cat# 25843-45-2, St. Louis, MO, USA). The recombinant mouse IGF1 (rIGF1) was purchased from R&D Systems (Cat#791-MG, Minneapolis, MN, USA). The mouse IGF1 ELISA kit was purchased from R&D Systems (Cat#SMG100, Minneapolis, MN, USA). The mouse CCL2 ELISA kit was purchased from R&D Systems (Cat#SMJE00, Minneapolis, MN, USA). The mouse Fractalkine ELISA kit was purchased from R&D Systems (Cat. #DY472, Minneapolis, MN, USA). IGF1R inhibitor PQ401 was purchased from R&D Systems (Cat#2768 Minneapolis, MN, USA). Mouse IGF-1 and fractalkine-neutralizing antibodies were purchased from R&D Systems (Cat#AF791 and AF472, Minneapolis, MN, USA). Gene-specific primers were purchased from commercial sources: *Igf1* (Cat# PPM03387F-200, Invitrogen, Waltham, MA, USA), *Ccl2* (Cat# 10025636, Bio-Rad, Hercules, CA, USA), *Cx3cl1* (Cat# PPM02959F-200, Invitrogen, Waltham, MA, USA), *Tnf-α* (Cat# PPM03113G-200, Invitrogen, Waltham, MA, USA), *Il-1β* (Cat# PPM03109F-200, Invitrogen, Waltham, MA, USA), *Il-6* (Cat# PPM03015A-200, Invitrogen, Waltham, MA, USA), and *Gapdh* (Cat# 10025636, Bio-Rad, Hercules, CA, USA).

### 4.2. Mouse Model

Male C57BL/6J mice were sourced from Charles River Laboratories (Wilmington, MA, USA). Sample size estimation was based on previous studies [17]. Experimental units were randomly assigned to control and treatment groups using a computer-generated randomization sequence. All animal work in the study was done with the approval of the University of Texas IACUC committee. The mice used in the study were housed under the following conditions: humidity, 30–70%; temperature, 72 °F; dark/light cycle, 12 h dark/12 h light. Mice received intracerebroventricular infusions of recombinant IGF1 (120 ng/day for 3 days) or saline through implanted cannulas (AP −0.34 mm, ML −1.00 mm, DV +2.00 mm relative to bregma) connected to osmotic minipumps (Alzet, Cupertino, CA, USA), as previously described [44]. After completion of the 3-day IGF1 or saline infusion, ALF and HE were induced in adult mice (20–25 g) by administering a single intraperitoneal dose of AOM (100 mg/kg body weight) toward the end of the light/dark cycle, according to established protocols. [45]. Control mice received an injection of an equivalent volume of saline (IP). Post-AOM treatment, mice were maintained on heating pads adjusted to 37 °C to prevent hypothermia. Rodents were provided with a standard diet and hydrogel on the cage floor to facilitate easy access to food and water. Modeling quality was assessed based on reproducible neurological decline, including progressive loss of reflexes (Figure 2), as well as consistent elevation of blood ammonia levels (Figure S3). The temporal progression of the disease was also monitored, with animals reaching advanced neurological stages within a predictable time frame following AOM administration. These criteria are consistent with previously established standards for AOM-induced acute liver failure [46].

The exact number of experimental units (individual mice) used in each group is indicated in the corresponding figure legends. Mice were used across all in vivo experiments. Sample sizes were determined based on previous studies using similar experimental paradigms and outcome measures. No formal a priori sample size calculation was performed.

Neurological and physiological monitoring was initiated 12 h following the AOM challenge and conducted at 2-h intervals. The azoxymethane (AOM)-induced acute liver failure model is a well-established and reproducible experimental model of hepatic encephalopathy in mice, characterized by progressive neurological decline and liver failure [18,47,48]. Expected adverse events associated with the AOM-induced hepatic encephalopathy model included dehydration and hypothermia, which were monitored throughout the experiment. No unexpected adverse events were observed. To minimize potential confounders, animals were randomly allocated to treatment groups, and the order of behavioral testing and sample collection was randomized. Cage positions were rotated regularly within the animal facility to minimize location effects. Measurements included body temperature, body weight, and a comprehensive neurological assessment encompassing pinna reflex, corneal reflex, tail flexion reflex, escape response, righting reflex, and ataxia. Each component was scored on a scale of 0 to 2, with higher scores indicating preserved reflexes. The overall neurological score at each time point represented the cumulative sum of all individual scores. Before the onset of coma, neurological status was categorized into three stages based on reflex assessments. The pre-neurological decline stage was defined by decreased overall activity in the absence of reflex impairments or ataxia. Mice were classified as exhibiting minor neurological decline when delays were observed in one or more reflex responses, accompanied by mild ataxia, such as difficulty traversing a metal cage lid. Major neurological decline was defined by pronounced deficits across all assessed reflexes, along with marked ataxia [18]. Animals were allocated to experimental groups by a designated investigator who was aware of group assignment. Behavioral testing and outcome assessment were performed by personnel blinded to group allocation. Data analysis was conducted in a blinded manner.

Two independent cohorts of mice were included in the study. In the first cohort, animals were monitored until the loss of both corneal and righting reflexes to determine the time to coma (complete loss of all reflexes), after which they were euthanized. In the second cohort, mice were euthanized at the appearance of mild neurological deficits (approximately 16 h post-AOM treatment) to allow tissue collection for the evaluation of early molecular and cellular alterations occurring before the development of severe HE. At the time of euthanasia, mice were administered Euthasol (120 mg/kg), followed by either bilateral thoracotomy for tissue harvesting for molecular analyses or transcardial perfusion with phosphate-buffered saline (PBS) followed by 4% paraformaldehyde in PBS for whole-brain collection and subsequent histological analyses.

### 4.3. ELISA Analysis

For protein extraction, the cortex, cerebellum, and hippocampus tissues were weighed and homogenized using a Miltenyi Biotec gentleMACS dissociator (San Diego, CA, USA). Homogenates were incubated on ice for 30 min and centrifuged at 16,000× *g* for 15–20 min at 4 °C. The supernatants were collected, and total protein concentration was measured using a ThermoFisher Pierce BCA Protein Assay kit (Cat#23225, Waltham, MA, USA). Samples were kept at −80 °C and underwent a single freeze–thaw cycle.

For mouse IGF1 kits, capture antibodies were incubated overnight in 96-well plates. After this, the assay was performed according to the instructions provided by R&D Systems. Each sample received either 100 μg of protein or 100 μL of undiluted conditioned cell media. Absorbance was read using a SpectraMax M5 plate reader from Molecular Devices (Sunnyvale, CA, USA). IGF1 concentration was expressed per milligram of total lysate protein.

For mouse CCL2 and fractalkine kits, the assay was performed according to the instructions provided by R&D Systems. For the CCL2 assay, HT-22 conditioned media were diluted 1:2 before loading, whereas undiluted conditioned media were used for fractalkine measurements. Absorbance was read using a SpectraMax M5 plate reader from Molecular Devices (Sunnyvale, CA, USA). Cytokine concentrations were calculated based on standard curves generated with recombinant standards provided in the kits.

### 4.4. Immunofluorescence

Whole brains were fixed in paraformaldehyde, cryoprotected in 30% sucrose, and sectioned for subsequent analysis. Free-floating immunofluorescence staining was conducted on 30 μm-thick brain sections obtained from the cortex. Sections were blocked with 5% goat serum and incubated with primary antibodies at 4 °C overnight against IGF1 (1:10, SC-9013, Santa Cruz Biotechnology, Dallas, TX, USA), IBA1 (1:100, Cat# SAB5701363, Millipore-Sigma, Burlington, MA, USA), NeuN (1:100, MAB377, Millipore Sigma, Burlington, MA, USA), or GFAP (1:100, MAB3402, Millipore Sigma, Burlington, MA, USA). Immunoreactivity was detected using Cy3- (1:100, Cat#715-167-003, Jackson ImmunoResearch, West Grove, PA, USA) or Alexa Fluor 488-conjugated secondary antibodies (1:100, Cat#711-545-152, Jackson ImmunoResearch, West Grove, PA, USA). Brain sections were positioned on positively charged slides, and coverslips were mounted with ProLong Gold Antifade Reagent containing DAPI (Cat#P36932, Thermo Fisher Scientific, Waltham, MA, USA). Slides were subsequently imaged using a Leica TCS SP5-X inverted confocal microscope (Leica Microsystems, Wetzlar, Germany).

### 4.5. Microglia, Astrocytes, and Neuron Isolation

Microglia, astrocytes, and neurons were isolated from mouse brains in sequence using a Miltenyi Biotec (Bergisch Gladbach, Germany) MACS-based cell separation system at 4 °C to improve cell viability and RNA integrity. Brain tissue was enzymatically and mechanically dissociated to obtain a single-cell suspension, following the instructions of Neural Tissue Dissociation Kits (Cat#130-092-628), followed by the removal of debris and myelin, according to the instructions provided by Myelin Removal Beads II (Cat#130-096-733).

Microglia were subsequently isolated by positive magnetic selection using CD11b MicroBeads (Cat#130-093-634) and MACS^®^ separation columns, following the manufacturer’s protocol. Briefly, CD11b^+^ cells were magnetically labeled, retained within the column under a magnetic field, and eluted as the positively selected microglial fraction.

The CD11b^−^ flow-through fraction was then subjected to astrocyte isolation using an Anti-GLAST (ACSA-1) MicroBead Kit (Cat# 130-095-825). GLAST^+^ astrocytes were magnetically labeled and positively selected according to the manufacturer’s instructions, yielding a purified astrocyte population.

The remaining unlabeled cell fraction following CD11b and GLAST magnetic separations was defined as the neuronal-enriched population and was collected for downstream molecular analyses.

### 4.6. Immunohistochemistry

Immunohistochemical staining was conducted on 30 μm thick cortical brain sections using a free-floating method. Sections were blocked in 5% goat serum and incubated overnight with a primary antibody against IBA1 (1:100, Cat#SAB5701363, Millipore-Sigma, St. Louis, MO, USA), followed by incubation with a goat anti-rabbit secondary antibody. Immunostaining was developed using 3,3′-diaminobenzidine [49] as the chromogen (Vector Laboratories, Newark, CA, USA). Sections were subsequently mounted onto positively charged slides, dehydrated through graded ethanol and xylene, and coverslipped with Cytoseal XYL. Images were acquired using a Leica Aperio AT2 Digital Pathology System.

Broad-field microglial analysis was conducted using ImageJ (version 1.53k; National Institutes of Health, Bethesda, MD, USA). For each brain region, counts from 10 representative images were averaged to obtain the mean microglial density per mouse. Images were converted to 8-bit grayscale and subjected to a uniform threshold (40–255), followed by background noise masking and removal of outlier particles (“radius = 3, threshold = 5, which = dark”). Despeckling was applied before particle analysis. Finally, IBA1-positive cell bodies were quantified using the Analyze Particles function in ImageJ to obtain cell body counts for each image. Microglial soma size was quantified in ImageJ using the freehand selection tool to manually outline individual cell bodies (a minimum of 300 cells per mouse), after which the selected areas were measured. Images were acquired using a 20× objective and calibrated in ImageJ before analysis. Soma area measurements from 10 randomly selected images per brain region were averaged to determine the mean soma size for each animal. Image analysis was performed by an investigator blinded to the experimental groups. Sholl analysis was performed on microglia (n = 50 cells per mouse) using ImageJ with the FIJI Simple Neurite Tracer (SNT) Neuroanatomy plugin, employing an initial radius of 3 pixels and incremental steps of 5 pixels, with the maximum radius set to 150 pixels.

### 4.7. Liver Histology and Serum Chemistry

Liver samples preserved in formalin and embedded in paraffin were sectioned to a 4 μm thickness and placed on positively charged slides (Santa Cruz Biotechnology, sc-363562, Santa Cruz, CA, USA). Sections were deparaffinized by sequential xylene washes and rehydrated through a graded ethanol series ranging from 100% to 80%. Liver sections were stained with hematoxylin QS for 1 min, rinsed in tap water, differentiated with 0.1% ammonium hydroxide, and subsequently counterstained with eosin Y for 4 min, followed by washing in deionized water. Sections were dehydrated using ascending concentrations of ethanol (95–100%) and cleared with three xylene washes. Coverslips were mounted using Cytoseal XYL mounting medium (Thermo Fisher Scientific, Waltham, MA, USA), and slides were imaged using a Leica Aperio AT2 Digital Pathology System (Deerfield, IL, USA).

Liver injury was assessed by quantifying serum aspartate aminotransferase (AST) and alanine transaminase (ALT) enzyme activities. All measurements were performed using assay kits following the manufacturer’s protocols (Sigma-Aldrich, St. Louis, MO, USA).

### 4.8. mRNA Analyses

Total RNA was isolated from frozen cortex samples using the RNeasy Mini Kit in accordance with the manufacturer’s instructions. RNA concentration and purity were determined with a NanoDrop 2000 spectrophotometer (Thermo Fisher Scientific, Waltham, MA, USA), followed by reverse transcription into cDNA using the iScript cDNA Synthesis Kit, as previously reported. Quantitative PCR was carried out using primers specific for IGF1, C-C motif ligand 2 (CCL2), fractalkine (CX3CL1), interleukin-1β (IL-1β), IL-6, tumor necrosis factor-α (TNF-α), and GAPDH (Table 1). Relative gene expression was calculated using the ΔΔCt method with CFX Maestro software (version 2.3; Bio-Rad Laboratories, Hercules, CA, USA), using control cortical tissues as reference groups, respectively [50].

### 4.9. Cell Culture

The mouse microglial cell line EOC-20 and the mouse hippocampal neuron cell line HT-22 were obtained from commercial sources and maintained in culture according to ATCC recommendations (Manassas, VA, USA). Cells were maintained in Dulbecco’s Modified Eagle Medium (DMEM; Gibco, Waltham, MA, USA) supplemented with 10% fetal bovine serum (FBS; Gibco, Waltham, MA, USA), 1% L-glutamine, and 1% penicillin–streptomycin (Thermo Fisher Scientific, Waltham, MA, USA). All cells were cultured at 37 °C in a humidified atmosphere containing 5% CO_2_. Cells within passages P10–P15 were used for all experiments. For in vitro experiments, HT-22 or EOC-20 cells were seeded at a density of approximately 1 × 10^5^ cells per well (or equivalent density depending on plate format) and allowed to adhere overnight. HT-22 cells were treated with rmIGF-1 (10 ng/mL) and the IGF1R inhibitor PQ401 (25 μM) for 4 h. HT-22 cells were washed with PBS to remove residual compounds and incubated with fresh culture medium. After 24 h, the cells were harvested for RNA isolation, followed by RT-PCR analysis, and conditioned media were collected. One portion of the conditioned media was directly used for ELISA to quantify fractalkine and CCL2 levels. A separate portion was incubated with either an IGF-1 or fractalkine-neutralizing antibody, clarified by centrifugation, and subsequently applied to EOC-20 microglial cells. EOC-20 microglial cells were then incubated for an additional 24 h to allow accumulation of neuron-derived factors in the conditioned medium. For phagocytosis measurements, EOC-20 cells were plated at a density of 50,000 cells per well in black 96-well culture plates. After cell attachment, EOC-20 cells were treated with one of the following conditions: (i) increasing concentrations of rmIGF-1 (1–10 ng/mL); (ii) conditioned media collected from HT-22 cells treated with increasing concentrations of rmIGF-1; or (iii) conditioned media derived from rmIGF-1-treated (10 ng/mL) HT-22 cells that had been incubated with either an IGF-1 neutralizing antibody (1.0 μg/mL) or a fractalkine-neutralizing antibody (1.5 μg/mL). Phagocytic activity of EOC-20 cells was then assessed using the Vybrant™ Phagocytosis Assay Kit (Cat# V6694, Thermo Fisher Scientific, Waltham, MA, USA) following the manufacturer’s instructions.

### 4.10. Statistical Analysis

The experimental unit for all behavioral assessments and biochemical measurements was a single mouse. For immunohistochemistry and microglial morphometric analyses, the experimental unit was an individual mouse. Multiple brain sections and images were analyzed per animal, and the mean value per mouse was used for statistical comparisons. For Western blot and ELISA analyses, the experimental unit was an individual mouse, with tissues collected and analyzed separately for each animal. For in vitro experiments, the experimental unit was an independent cell culture well. Each experiment was repeated using independently prepared cultures.

Data are presented as the mean ± standard error of the mean (SEM). Statistical analysis was performed with GraphPad Prism software (v10.2, San Diego, CA, USA). Normality was assessed using the Shapiro–Wilk test, and homogeneity of variances was evaluated using the Brown–Forsythe test. The difference between the two groups was analyzed using a two-tailed *t*-test. For normally distributed data with equal variances, differences among multiple groups were analyzed by ordinary one-way ANOVA followed by Tukey’s post hoc test. When variances were unequal, Brown–Forsythe and Welch one-way ANOVA were applied with Games–Howell multiple comparison post hoc testing. For data that were not normally distributed, comparisons among multiple groups were performed using the Kruskal–Wallis test followed by Dunn’s multiple comparison test. A *p*-value < 0.05 was statistically significant. The differences in Sholl profiles among groups were analyzed using two-way ANOVA, with Factor A (treatment group) and Factor B (radius), followed by Tukey’s post hoc multiple comparison test.

## 5. Conclusions

In summary, our data indicate that AOM-induced HE is accompanied by a rapid, region-selective suppression of cortical IGF1 signaling, which is associated with maladaptive microglial phenotypic remodeling and a selectively altered neuroinflammatory program. Central restoration of IGF1 signaling via rmIGF1 infusion mitigates these CNS changes—normalizing microglial morphology/complexity and modulating key chemokine and cytokine expression—without significantly altering the severity of underlying liver injury, supporting a functional dissociation between hepatic damage and neurological outcomes in this model. Together, these findings suggest that impaired central IGF1 signaling is an important contributor to microglia-driven neuroinflammation and neurological dysfunction during acute HE and that therapeutic strategies aimed at augmenting brain IGF1 signaling may represent a viable adjunct approach for acute HE management.

## Figures and Tables

**Table 1:** Primer manufacturer and sequence information.

Primer	RefSeq Position	Sequence Amplified (3′-5′)
*Igf1*	585 (NM_010512)	
*Ccl2*		3′-CCGTAAATCTGAAGCTAATGCATCC ACTACCTTTTC CACAACCACCTCAAG CACTTCTGTAGGAGTGACCAGTGTGA CAGTGAACTAGTGTGACTCGGACTGT GATGCCTT-5′
*Cx3cl1*	1029 (NM_009142)	
*Tnf-* *α*	194 (NM_013693)	
*Il-* *1β*	438 (NM_008361)	
*Il-6*	120 (NM_001314054)	
*Gapdh*		3′-TGGGAGTTGCTGTTGAAGTCGCAGG AGACAACCTGGTC CTCAGTGTAGCCC AAGATGCCCTTCAGTGGGCCCTCAGAT GCCTGCTTCACCACCTTCTTGATGTCA-5′

## Data Availability

The raw data supporting this study have been deposited in Figshare and are publicly available at https://doi.org/10.6084/m9.figshare.31892272.

## Supplementary Materials

The following supporting information can be downloaded at: https://www.mdpi.com/article/10.3390/ijms27083547/s1.

## Abbreviations

The following abbreviations are used in this manuscript:

ALF	Acute liver failure
ALT	alanine aminotransferase
AST	aspartate aminotransferase
AOM	azoxymethane
CCL2	C–C motif chemokine ligand 2
CNS	central nervous system
CX3CL1	C–X3–C motif chemokine ligand 1 (fractalkine, FKN)
IGF1	insulin-like growth factor 1
IGF1R	insulin-like growth factor 1 receptor
IL-1β	interleukin-1 β
IL-6	interleukin-6
rmIGF1	recombinant mouse IGF1
TNF-α	tumor necrosis factor-α
