# Supplementary material for: Central Administration of Recombinant IGF1 Is Neuroprotective in a Rodent Model of Acute Liver Failure
Source: Int J Mol Sci. 2026 Apr 16;27(8):3547. doi: 10.3390/ijms27083547 (PMC13116921; doi:10.3390/ijms27083547)
Supplement: Supplementary file 1 [file ijms-27-03547-s001.zip › ijms-4101987-supplementary.pdf]

Article

Central administration of recombinant IGF1 is neuroprotective in a rodent model of acute liver failure

Yubo Wang [1], Matthew McMillin [2], Gabriel Frampton [3], Kathryn Rhodes [1], Elaina Williams [1], Juliet Venter [1], Jace Tyson [1], Esha Gupta [1], Mihika Patankar [1], Patrick Mireles [1] and Sharon DeMorrow [4,*]

[1] Division of Pharmacology and Toxicology, College of Pharmacy, The University of Texas at Austin, Austin, Texas, USA; yubowang@utexas.edu (Y.W.); kathryn.rhodes@utexas.edu (K.R.); elainacoree@gmail.com (E.W.); Juliet.venter@austin.utexas.edu (J.V.); jace.e.tyson@utexas.edu (J.T.); eshagupta@utexas.edu (E.G.); mihikapatankar@utexas.edu (M.P.); pr_mireles@utexas.edu (P.M.)

[2] Baylor College of Medicine, Department of Innovation, Education and Technology, Temple, Texas, USA; Matthew.McMillin@bcm.edu (M.M.)

[3] Department of Neurosurgery, Dell Medical School, The University of Texas at Austin, Austin, Texas, USA; Gabriel.Frampton@austin.utexas.edu (G.F.)

[4] Division of Pharmacology and Toxicology, College of Pharmacy, Department of Internal Medicine, Dell Medical School, The University of Texas at Austin, Austin, Texas, USA.

* Correspondence: Sharon.demorrow@austin.utexas.edu; Tel.: +1 512 4955779

Supplemental figures

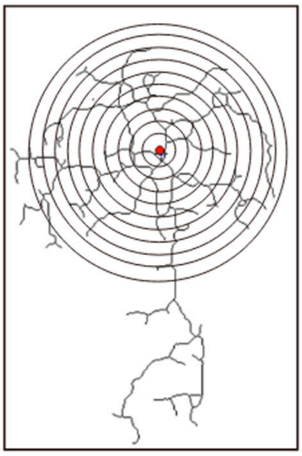

Figure S1. Schematic illustration of Sholl analysis for microglia. A representative reconstructed microglial cell is shown with concentric circles centered on the soma (red dot). Sholl profiles were generated by counting the number of intersections between microglial processes and each circle as a function of radial distance from the soma, providing a quantitative readout of process ramification.

.

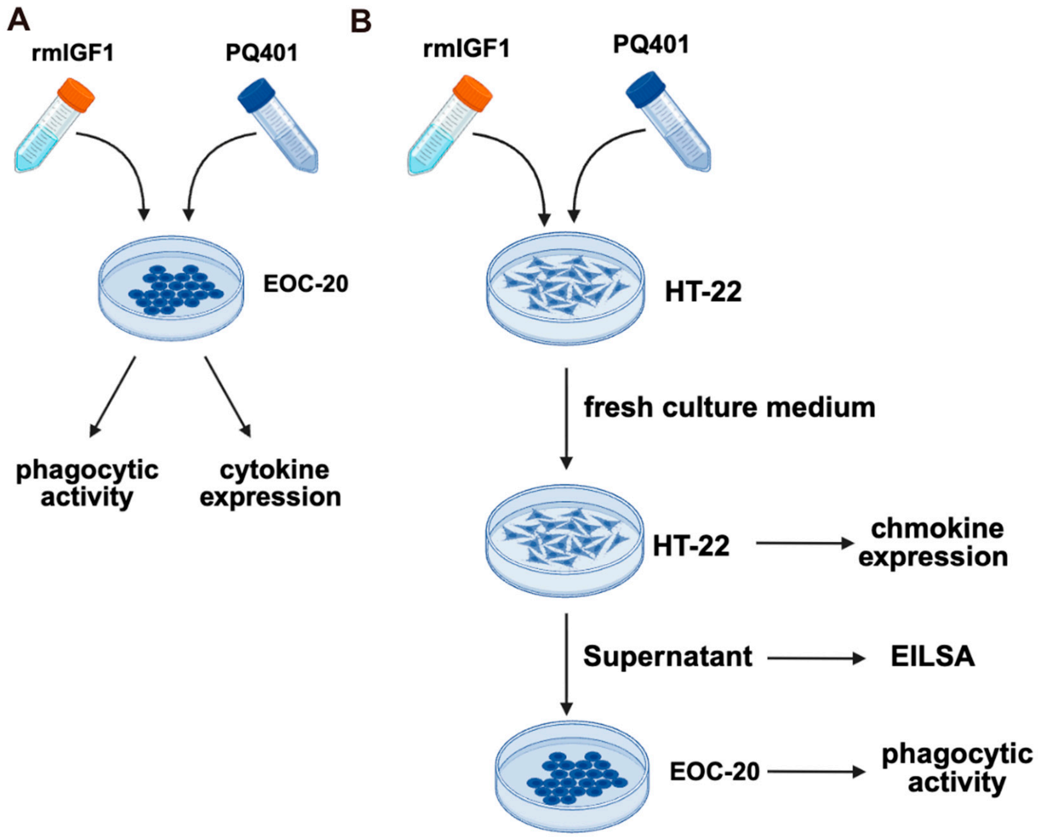

Figure S2. Schematic illustration of in *vitro* paradigms used to assess direct and neuron-mediated effects of rmIGF1 on microglial readouts. (A) EOC-20 microglial cells were treated with rmIGF1 (1 μg/mL) and PQ401 (25 μM) for 4 hours and subsequently assayed for phagocytic activity and inflammatory cytokine gene expression by qPCR; (B) HT-22 neuronal cells were treated with rmIGF1 and/or the PQ401 for 4 h. After treatment, the medium was removed and replaced with fresh culture medium. HT-22 cells were then incubated for an additional 24 h to generate conditioned medium. Chemokine expression in HT-22 cells was analyzed, and the collected conditioned medium was used for ELISA measurement of secreted factors. The conditioned medium was subsequently applied to EOC-20 microglial cells to assess its effects on microglial phagocytic activity.

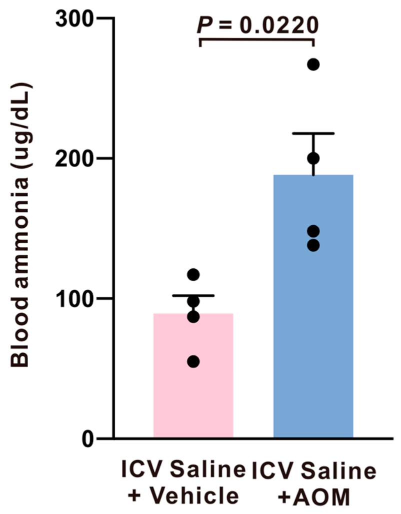

Figure S3. AOM administration increases blood ammonia levels. Blood ammonia levels were measured in mice receiving ICV saline, followed by either vehicle or AOM administration. AOM-treated mice exhibited significantly elevated blood ammonia levels compared to vehicle controls. Data are presented as mean ± SEM with individual data points shown. Statistical analysis was performed using an unpaired two-tailed Student's *t*-test.
